# Breast Cancer Cells Reprogram the Oncogenic lncRNAs/mRNAs Coexpression Networks in Three-Dimensional Microenvironment

**DOI:** 10.3390/cells11213458

**Published:** 2022-11-01

**Authors:** Stephanie I. Nuñez-Olvera, Lorena Aguilar-Arnal, Mireya Cisneros-Villanueva, Alfredo Hidalgo-Miranda, Laurence A. Marchat, Yarely M. Salinas-Vera, Rosalio Ramos-Payán, Carlos Pérez-Plasencia, Ángeles Carlos-Reyes, Jonathan Puente-Rivera, Cesar López-Camarillo

**Affiliations:** 1 Departamento de Biología Celular y Fisiología, Instituto de Investigaciones Biomédicas, Universidad Nacional Autónoma de México, Mexico City 04510, Mexico; 2 Laboratorio de Genómica del Cáncer, Instituto Nacional de Medicina Genómica, Mexico City 14610, Mexico; 3 Programa en Biomedicina Molecular y Red de Biotecnología, ENMyH-Instituto Politécnico Nacional, Mexico City 07320, Mexico; 4 Departamento de Bioquímica, Centro de Investigación y Estudios Avanzados, Mexico City 07360, Mexico; 5 Facultad de Ciencias Químico Biológicas, Universidad Autónoma de Sinaloa, Culiacán 80040, Mexico; 6 Laboratorio de Genómica, Instituto Nacional de Cancerología (INCan). Av. San Fernando 22, Col. Sección XVI. Tlalpan, Mexico City 14080, Mexico; 7 Laboratorio de Genómica, Unidad de Biomedicina, FES-Iztacala, Universidad Nacional Autónoma de México, Tlalnepantla 54090, Mexico; 8 Laboratorio de Onco-Inmunobiología, Departamento de Enfermedades Crónico-Degenerativas, Instituto Nacional de Enfermedades Respiratorias (INER) Ismael Cosio Villegas, Mexico City 14080, Mexico; 9 División de Ciencias de la Salud, Biológicas y Ambientales, Universidad Abierta y a Distancia, Mexico City 03330, Mexico; 10 Posgrado en Ciencias Genómicas, Universidad Autónoma de la Ciudad de México, Mexico City 03100, Mexico

**Keywords:** breast cancer, organotypic 3D cultures, lncRNAs, co-regulation networks, therapy response

## Abstract

Organotypic three-dimensional (3D) cell cultures more accurately mimic the characteristics of solid tumors in vivo in comparison with traditional two-dimensional (2D) monolayer cell models. Currently, studies on the regulation of long non-coding RNAs (lncRNAs) have not been explored in breast cancer cells cultured in 3D microenvironments. In the present research, we studied the expression and potential roles of lncRNAs in estrogen receptor-positive luminal B subtype BT-474 breast cancer cells grown over extracellular matrix proteins-enriched 3D cultures. Global expression profiling using DNA microarrays identifies 290 upregulated and 183 downregulated lncRNAs in 3D cultures relative to 2D condition. Using a co-expression analysis approach of lncRNAs and mRNAs pairs expressed in the same experimental conditions, we identify hundreds of regulatory axes modulating genes involved in cancer hallmarks, such as responses to estrogens, cell proliferation, hypoxia, apical junctions, and resistance to endocrine therapy. In addition, we identified 102 lncRNAs/mRNA correlations in 3D cultures, which were similar to those reported in TCGA datasets obtained from luminal B breast cancer patients. Interestingly, we also found a set of mRNAs transcripts co-expressed with LINC00847 and CTD-2566J3.1 lncRNAs, which were predictors of pathologic complete response and overall survival. Finally, both LINC00847 and CTD -2566J3.1 were co-expressed with essential genes for cancer genetic dependencies, such as FOXA1 y GINS2. Our experimental and predictive findings show that co-expressed lncRNAs/mRNAs pairs exhibit a high degree of similarity with those found in luminal B breast cancer patients, suggesting that they could be adequate pre-clinical tools to identify not only biomarkers related to endocrine therapy response and PCR, but to understand the biological behavior of cancer cells in 3D microenvironments.

## 1. Introduction

Breast cancer is highly heterogeneous, with distinctive genotypic, phenotypic, and anatomical characteristics that influence each patient’s clinical outcome [1]. Although their histological description and molecular classification may appear simple, this heterogeniety has encouraged biological studies of molecular subtypes in order to understand the biological behavior of each tumor and develop therapeutic strategies. Currently, knowledge about breast carcinomas comes from in vivo and in vitro research mostly supported by cancer cell lines classified into molecular subtypes [2,3,4]. There are around 84 breast cancer cell lines classified according to the presence of four major molecular subtypes: human epidermal growth factor receptor 2 (HER2)-enriched, basal-like, luminal A, and luminal B [5]. The luminal B subtype classification describes ER+ cancers with low progesterone receptors expression, high proliferation, and a poor response to hormonal therapy when contrasted to luminal A [6,7]. Additionally, it shows aggressive clinical behavior and has a prognosis comparable to non-luminal tumors (HER2 and basal-like) [7,8].

Interestingly, a large part of the available research on the B subtype has been performed using the BT-474 cell line mostly cultured in monolayer. Notably, in the BT-474 cell line, expression of the three receptors ER+, PR+, and HER2+ is detected, hereby bridging the gap between the luminal and non-luminal breast cancer subtypes, and as such, constituting a relevant model for both in vivo and in vitro studies [5].

Cell cultures are essential and crucial procedures in drug development and basic mechanisms of cancer research [9]. Most cells are cultivated using 2D methods; however, novel approaches that employ 3D cell culturing techniques provide persuasive evidence to generate important advances in cancer research [10,11]. Different 3D cell culture models, such as organoids, organotypic, and spheroids, may be generated using particular methods. Spheroids are multicellular units that are cultivated as free-floating aggregates in low attachment plastic plates. On the other hand, organoids and organotypic cultures use extracellular matrix (ECM) compounds as scaffolds to grow in 3D structures. Organoids are tumor cell cultures derived from individual tumor biopsies from patients, which are obtained by surgical procedures. On the other hand, organotypic cultures are obtained by culturing cancer cell lines in ECM used as platform for growth in 3D structures. These cell cultures are considered to mirror human tumors more realistically than cells grown as a 2D monolayer on plastic surfaces [12,13]. The tumor microenvironment modulates tumorigenesis through the ECM and the diverse cellular lineages [14]. The cell environment may be adjusted in 3D cell culture procedures to imitate in vivo conditions and offer more precise data regarding cell-to-cell interactions, tumor morphological and molecular features, drug discovery, metabolic profiling, and stem cell research [15,16]. The morphogenesis, invasiveness, and metastatic capabilities of breast cancer cells grown in 3D cultures are intimately associated with their gene expression profiles [17]. Furthermore, gene expression in 3D cultures appears to be more similar to clinical tumor tissues, which has been attributed, at least in part, to characteristics such as the formation of different cell layers and cell-cell/ECM-cell connections, allowing the formation of distinct microenvironments. These conditions generate hypoxia and nutrient deficiency in the core of 3D cultures [18,19,20]. Moreover, these physical properties can trigger signaling cascades affecting gene expression and tumor clinical behavior. 

Long non-coding RNAs (lncRNAs) have recently been described as non-coding RNAs (ncRNAs) controlling gene expression [21]. The significance of lncRNAs stems mostly from their capacity to form DNA, RNA, or protein complexes, and hence, influence gene expression at the transcriptional and post-transcriptional levels [22]. Furthermore, lncRNAs deregulation has been linked to a number of human illnesses, including cancer [21]. LncRNAs are single-stranded ncRNAs longer than 200 nucleotides which are transcribed by RNA polymerase II [21,23,24]. Several mechanisms such as serving as microRNAs sponges, signals, decoys, scaffolds, and guides, through which lncRNAs influence gene expression, have been postulated [25,26]. Numerous lncRNAs are expressed in close correlation with mRNAs involved in cancer hallmarks, such as proliferation, cell cycle regulation, apoptosis, cell migration, metastasis, and immune response in different types of tumors [27,28,29]. However, the study of lncRNAs/mRNAs landscapes in 3D cell cultures is still underexplored. So far, the few studies of breast cancer cell lines cultured in 2D versus 3D have been focused on exploring morphological and phenotypic characteristics or chemotherapy resistance, with just a minority of studies examining changes in ncRNAs expression of 3D cultures from cancer cell lines [30,31,32]. Here, we found significant differences in mRNA expression from BT-474 cells grown either in 3D microenvironments or in 2D monolayers, coincident with previous reports for other cancer cell lines [33]. Data showed the existence of significant differences in mRNA gene expression profiling in BT-474 cells grown in 3D microenvironments relative to 2D culture conditions. The co-regulatory networks analysis between lncRNAs/mRNAs pairs in 3D culture of BT-474 cells demonstrated that these pairs are significantly enriched in cancer hallmarks such as estrogen response, hypoxia, apical junction and therapy resistance. In addition, the correlated lncRNAs/mRNAs pairs were similar to those reported in TCGA for luminal B breast cancer enriched in cancer hallmarks. Specifically, we identified a set of mRNAs that co-expressed with the lncRNAs LINC00847 and CTD -2566J3.1 with a robust value as predictors of pathologic complete response and essential genes for cancer genetic dependency, which supports the use of 3D culture as a more useful pre-clinical model for the selection of therapeutic targets and/or biomarkers associated with specific molecular subtypes of breast cancer.

## 2. Materials and Methods

### 2.1. Generation of 3D Cultures from BT-474 Cells

BT-474 cell lines were obtained from ATCC (Manassas, Virginia) and cultured in Hybri-Care medium and 10% fetal bovine serum (FBS). For the generation of the 3D cultures, 120 µL of Geltrex (Thermo Fisher Scientific) was added to the 24-well plates and incubated at 37 °C for 30 min to allow solidification. Subsequently, 3.1 × 10^4^ cells from the 2D culture of BT-474 were added with 250 µL of Hybri-Care medium and incubated at 37 °C for 30 min. Finally, 250 µL of Hybri-Care medium supplemented with 5% matrigel was added, and the next day and every three days thereafter, the 3D cultures received 500 µL of fresh complete medium.

### 2.2. RNA Isolation from BT-474 Cells in 2D Monolayer and 3D Cultures

The total RNA for the analysis of lncRNAs and mRNAs expression by microarray was isolated from monolayer and 3D cultures of BT-474 at six days of culture, using cultures initiated with 3.1 × 10^4^ cells and obtaining a pool of 58 ± 1.34 spheroids over a period of 6 days. 1 mL of Trizol was added to BT-474 cells grown in 2D and 3D systems and mixed by pipetting, the cell lysate was recovered in a 1.5 mL Eppendorf tube, incubated for 5 min at RT, 200 µL of chloroform for each mL of Trizol reagent and mixed by vigorous shaking for 15 s. The tube was incubated for 3 min at RT and centrifuged at 12,500 rpm for 25 min at 4 °C. After centrifugation, the aqueous phase was recovered, taking care not to mix the other phases, and placed in a new 1.5 mL eppendorf tube. Then, 500 µL of isopropanol was used to precipitate RNA and the pellet was incubated on ice for 20 min. The tubes were centrifuged at 12,500 rpm for 25 min at 4 °C. The supernatant was removed and the pellet was washed with 1mL of 75% ethanol and allowed to dry for 5 min. Subsequently, the pellet was resuspended in 20 µL of nuclease-free water. Finally, the RNA obtained was quantified by spectrophotometry and visualized using 1% agarose/TAE 1× electrophoresis. 

### 2.3. Microarray Assays

The Clariom™D Assay Human (Affymetrix GeneChip, Santa Clara, CA, USA) was used to examine the expression profiles of lncRNAs, using biological triplicates in each condition of 2D and 3D cultures (n = 3). The Clariom array has 55,900 lncRNA probes and 18,858 mRNA gene probes. The microarray experiments were performed in accordance with the manufacturer’s instructions. In brief, 100 ng of total RNA were used to synthesize first-strand cDNA. After, DNA polymerase I and RNase H were employed to degrade the RNA while also synthesizing second-strand cDNA. Finally, the sense-strand cDNA was generated by reverse transcription and loaded into Clariom D arrays for human samples (Thermo Fisher Scientific, Waltham, MA, USA) and incubated for 16 h at 45 °C on the GeneChip Hybridation Oven 645 (Affymetrix Inc., Santa Clara, CA, USA). After hybridization, the arrays were washed and stained using the GeneChip Fluidics Station 450, followed by scanning with the GeneChip Scanner 3000 7G (Affymetrix Inc., Santa Clara, CA, USA). Raw data were analyzed using Affymetrix Expression Console and Transcriptome Analysis Console software. Differentially expressed genes were computed with the limma package by a moderate *t*-test and adjusted *p*-value by FDR method. lncRNAs with ≥2-fold-change (FC) and *p* < 0.05 were selected as being significantly differentially expressed. The data was uploaded to the GEO omnibus with the following accession number (GSE206836).

### 2.4. LncRNA/mRNA Correlation Analysis

For all potential lncRNA/mRNA pairs, Pearson’s correlation coefficient was determined using R studio. If the absolute correlation score was >0.99 and the *p* value was 0.05, the pair was regarded as substantially co-expressed. Next, we searched for each lncRNA and downloaded the lncRNA/mRNA correlation data for luminal B breast cancer samples from TCGA in TANRIC [34] and lncSEA [35]. The threshold of Pearson’s correlation coefficients was set to >0.3 from TCGA data and >0.9 from 3D culture data.

### 2.5. GO and Pathway Analysis of DEGs

The mRNAs transcripts significantly up/down-regulated in co-expression with lncRNAs were subjected to gene ontology (GO) and pathway analyses for functional and characteristic classification of enriched genes through the Enrichment analysis online tool [36]. The top of GO terms and pathways were sorted according to their *p* values.

### 2.6. Gene Set Enrichment Analysis (GSEA)

To identify cancer hallmarks significantly enriched in 3D culture compared to 2D culture, gene set enrichment analysis (GSEA) was applied. For GSEA, fgsea R package was used with molecular signature database (MSigDB v.6.2) of the following gene sets: hallmark cancer gene sets and endocrine therapy resistance. Significantly enriched processes were defined based on an FDR < 0.1 and absolute normalized enrichment score (NES) > 1.0. Cytoscape (v.3.7) was used to visualize significantly enriched GSEA processes (FDR < 0.01; NES ± 1.0).

### 2.7. Analysis of Genetic Dependencies

Whole-genome RNAi screening data for Cancer Cell Lines Encyclopedia (CCLE) were generated in Project Achilles [37] (https://depmap.org/R2-D2/, accessed on 19 April 2022). The dependency scores obtained for the BT-474 cell line were used for analysis in this study. A normalized dependency score lower than −0.5 indicates a significant effect of RNAi knockdown.

### 2.8. Kaplan–Meier Analysis and ROC Curves

The prognostic roles of lncRNAs on overall survival were examined with Kaplan–Meier curve analysis with GEPIA and lnCAR, and the log-rank test was conducted to distinguish survival time, *p* < 0.05 was considered to indicate a statistically significant difference [38,39] in 104 and 192 samples respectively. Expression validation and predictive markers of mRNAs in co-expression with lncRNAs were analyzed using the transcriptome mRNA data of 404 luminal B samples and used to estimate the Receiver Operating Curve (ROC) in on-line plotter platform [40] through combined selection parameters such as luminal B breast cancer or HER2 and ER positive status.

## 3. Results

### 3.1. Generation and Morphological Characterization of 3D Organotypic Cultures of BT-474 Cells

**Figure 1:**
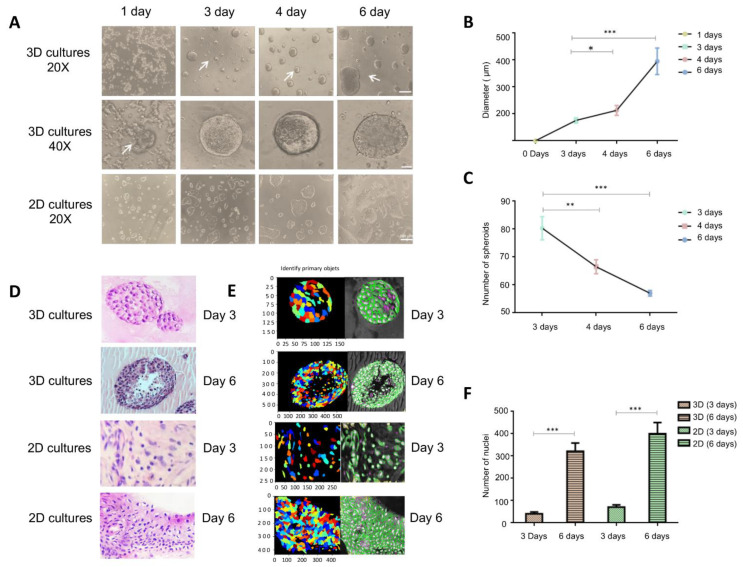
Generation and morphology of 3D organotypic cultures. (**A**) Growth kinetics of BT-474 cancer cells grown in 2D monolayers and 3D cell cultures observed in optical microscopy (20× and 40×) during 0–6 days incubation over Geltrex. (**B**) Quantification of the diameter of round-like structures in 3D cell cultures. (**C**) Quantification of the number of structures in 3D cultures at optimized seeding densities of 25,000 cells/well plate. (**D**) Hematoxylin and eosin staining of BT-474 cancer cells grown in 2D and 3D culture conditions. (**E**) Color image of 2D monolayers and 3D cell cultures using Identify Primary Objects module. Filter Objects module discards objects that have irregular shape or cell debris (magenta lines). (**F**) Quantification of number of nuclei present in 3D round-like structures. Values represent the mean ± SD from 3 independent experiments, using an unpaired Student’s *t*-test with unequal variances; * *p* < 0.05, ** *p* < 0.01, *** *p* < 0.001.

In order to determine the effects of 3D culturing on the lncRNAs landscape of BT-474 breast cancer cells corresponding to the luminal B subtype, we first set-up an on-top model for organotypic 3D cell culture formation by using as scaffold a commercial basement (Geltrex) reagent constituted by ECM proteins including laminin, collagen IV, entactin, and heparin sulfate proteoglycans, as described in materials and methods. Subsequently, we characterized the 3D cell cultures by tracking their growth kinetics over the course of 0 to 6 days. Results showed that 3D cultures were characterized by the formation of round-mass-like structures which initiated from day 3 until day 6 of incubation over the Geltrex (Figure 1A). During the kinetics, the 3D cell structures matured and significantly increased in size but decreased in number (Figure 1B,C), which may be explained by fusion events between round-like structures similar to those of water droplets, as previously described in both MCF-7 and MDA-MB-231 breast cancer cells [41]. To better characterize the morphology of spheroid-like structures, we performed histological sections and stained with hematoxylin and eosin to quantify the number of cell nuclei and internal distribution. Data indicated that at day 6, the number of cell nuclei significantly increased in comparison to day 3 (Figure 1D–F). Furthermore, a core free of cells was observed from day 3, which became more evident at day 6 (Figure 1E). This might be due to the cells’ heterogeneous dispersion resulting from the gradients of oxygen and nutrients in the internal layers of the 3D structures, as previously suggested [41]. Then, we analyzed the images using a tool to identify primary objects in 3D cultures in order to quantify nuclei per staining intensity and to remove objects that had irregular shape or cell debris. Results support the notion that the 3D structures have a diverse cellular distribution from day 3 until day 6 (Figure 1D,E). 

### 3.2. Identification of Differentially Expressed lncRNAs in 3D Cultures Derived from BT-474 Cell Line

**Figure 2:**
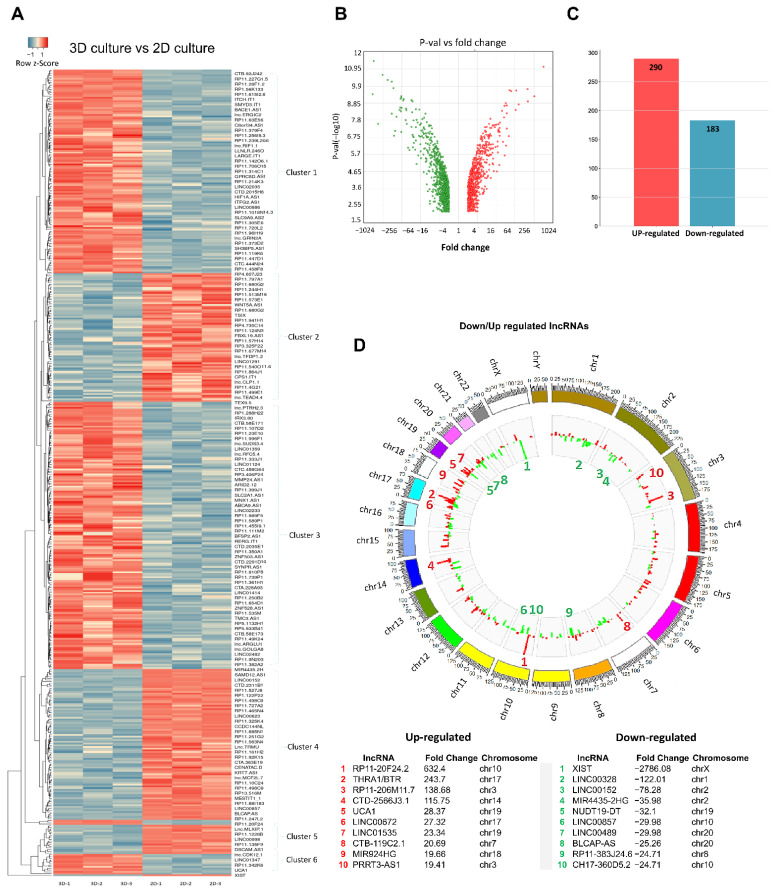
Expression profile of lncRNAs in 2D monolayers and 3D organotypic cell cultures. (**A**) Heatmap showing the hierarchical clustering of modulated lncRNAs by average linkage based on Euclidean distance measurements. Red represents down-regulation and green represents up-regulation. (**B**,**C**) Volcano and bar plots show differentials expressed lncRNAs in 3D vs. 2D cultures. Clusters of co-expressed genes are indicated. (**D**) Circos Plot analysis of chromosomal distribution of lncRNAs. Up-regulated lncRNAs are denoted in red bars and downregulated lncRNAs are shown in green bars in the gray internal circle. Height indicates degree of lncRNAs differential expression. Boton list show the chromosomal location of the top-ten deregulated lncRNAs in 3D cultures.

To identify the global expression profiles of lncRNAs in BT-474 cancer cells grown in 2D monolayers and 3D cell cultures, the Clariom™D Assay was used. 3D cell cultures were implemented, and at 6 days, total RNA was purified and submitted to microarrays analysis. Data showed that 473 lncRNAs were significantly modulated in 3D cell cultures, 290 of which were up-regulated and 183 down-regulated (fold change value > 2.0 or −2.0; *p* value 0.05 as cut-off values) (Figure 2A–C). Then, we used a two-track circus plot to determine the distribution of lncRNAs genes at the genomic level (Figure 2D). Data showed that the greatest number of up-regulated lncRNAs were located at 3, 12, 17, and 19 chromosomes. For instance, RP11-206M11.7, PRRT3-AS1, THRA, LINC00672, UCA1, and LINC01535 up-regulated lncRNAs were located on chromosomes 3, 17, and 19, respectively. On the other hand, the highest down-regulated lncRNAs were detected most frequently on 1, 7, and 17 chromosomes. According to earlier research, the highly aneuploid cell line BT-474 has a modal number of chromosomes that is similar to tetraploidy. It is interesting to note that BT-474 exhibited a partial chromosomal X deletion [42,43]. The low proportion of lncRNAs with differential expression (up/down regulated) discovered on the X chromosome as XIST in 3D cultures may be explained by this. Similarly, 91% of BT-474 cell line [42], where a low fraction of lncRNAs was also obtained, have been reported to have loss of chromosome 22. Gain of certain chromosomes, including 7, 12, and 17, has been identified in BT-474 cells [42] and may be related to increase of the number of lncRNAs with differential expression in 3D cultures located on chromosomes 12 and 17.

Then, we analyzed the reported functions of the top-ten lncRNAs modulated in 3D cultures. Interestingly, some of the lncRNAs with the highest fold change values, such as RP11-20F24.2, UCA1, LINC00672 (up-regulated); and XIST, CYTOR, LINC00857, MIR4435-2HG (down-regulated), have been previously described as deregulated in different types of human cancers with functions associated to cell growth and proliferation, migration, invasion, and metastasis [44,45,46,47,48,49,50,51,52] (Table 1 and Table 2). However, the functions of a number of lncRNAs, such as RP11-206M11.7, RP11-782C8.3, and RP11-425M5.7, among others, remain still unknown.

### 3.3. Establishment of Co-Expressed mRNAs/lncRNAs Pairs and Analysis of Biological Functions

**Figure 3:**
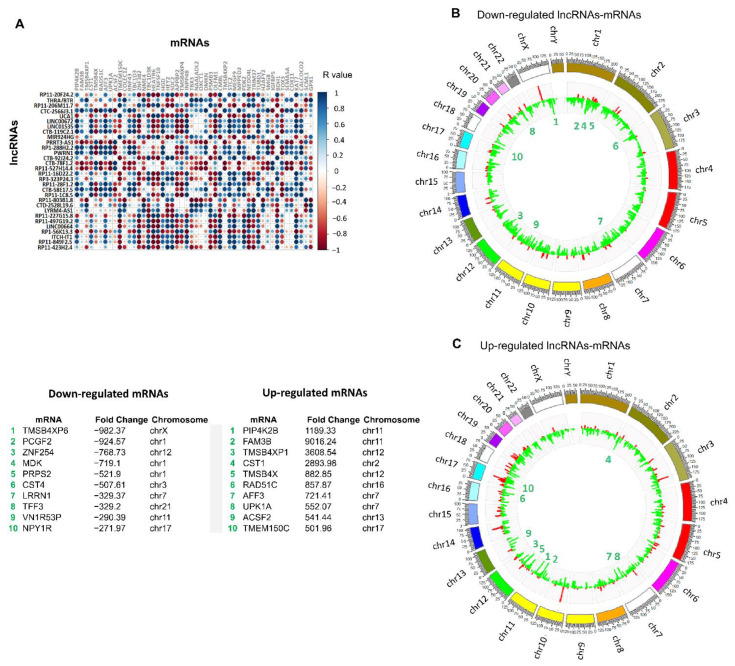
Correlated mRNAs/lncRNAs pairs. (**A**) Representative plot of the Pearson’s correlations found in the first 30 lncRNAs with the highest values of fold change. Blue circles represent the over-expressed lncRNAs/mRNAs pairs with a degree of positive linear association (≥0.9). (**B**) Circos plot of chromosomal distribution of down-regulated lncRNAs/mRNAs correlated pairs, lncRNAs are shown in red and mRNAs in green. Height indicates degree of differential expression. (**C**) Circos plot of chromosomal distribution of up-regulated lncRNAs/mRNAs correlated pairs; lncRNAs are shown in red and mRNAs in green. Height indicates degree of differential expression. Bottom list indicates the chromosomal location of the top-ten deregulated lncRNAs in 3D cultures.

LncRNAs are functionally involved in several biological processes; however, they are not represented in databases such as KEGG or gene ontology (GO), this makes it difficult to predict the biological processes and pathways in which they are involved. However, the identification of protein-coding genes that are co-expressed with lncRNAs can help to assign biological functions to lncRNAs [53,54,55]. To do this, we used the expression dataset from 4799 mRNAs modulated in BT-474 cell cultures in 3D conditions relative to 2D cultures (our unpublished data) to measure correlations between expressions of lncRNAs/mRNA pairs using Pearson’s correlation (Supplementary Data S1), as previously reported [53,54,55]. Correlation analyses were performed between the group of 290 lncRNAs and 1924 mRNAs over-expressed in 3D, and the group of 183 lncRNAs and 2875 mRNAs down-regulated in 3D cultures to identify lncRNAs/mRNAs pairs in co-expression. A positive or negative R value ≥ 0.99 and a *p* value < 0.05 were used as cut-off points, obtaining a total of 2906 correlations in both groups, within which, 1642 correlations were obtained between pairs of down-regulated mRNAs/lncRNAs (156 unique lncRNAs) and 1293 correlations in the pairs of over-expressed mRNAs/lncRNAs (284 unique lncRNAs). Figure 3A shows the correlations of the 30 most over-expressed lncRNAs in 3D cultures. In the intense blue dots, the over-expressed lncRNAs/mRNAs pairs are shown with positive linear association (R ≥ 0.9), which indicates a very high correlation between the lncRNAs/mRNAs pairs, in other words, when the expression of a lncRNA increases, the expression of the corresponding mRNA also does, following comparable magnitudes. On the contrary, the red dots show negative correlations; that is, while the expression of the lncRNA decreases, the value of the associated mRNA also decreases. Finally, up- and down-regulated lncRNA/mRNA pairs correlated are displayed in a circos plot to determine the chromosomal distribution. Data showed that down-regulated lncRNAs and mRNAs (Figure 3B) have a homogeneous distribution on chromosomes 1 and 16. Furthermore, overexpressed mRNAs and lncRNAs have a greater density on chromosomes 17 and 19, which contain numerous well-known oncogenes including *HER2*, *TOP2A*, and *TAU*, as well as tumor suppressor genes like *p53*, *BRCA1*, and *HIC-1*.

**Figure 4:**
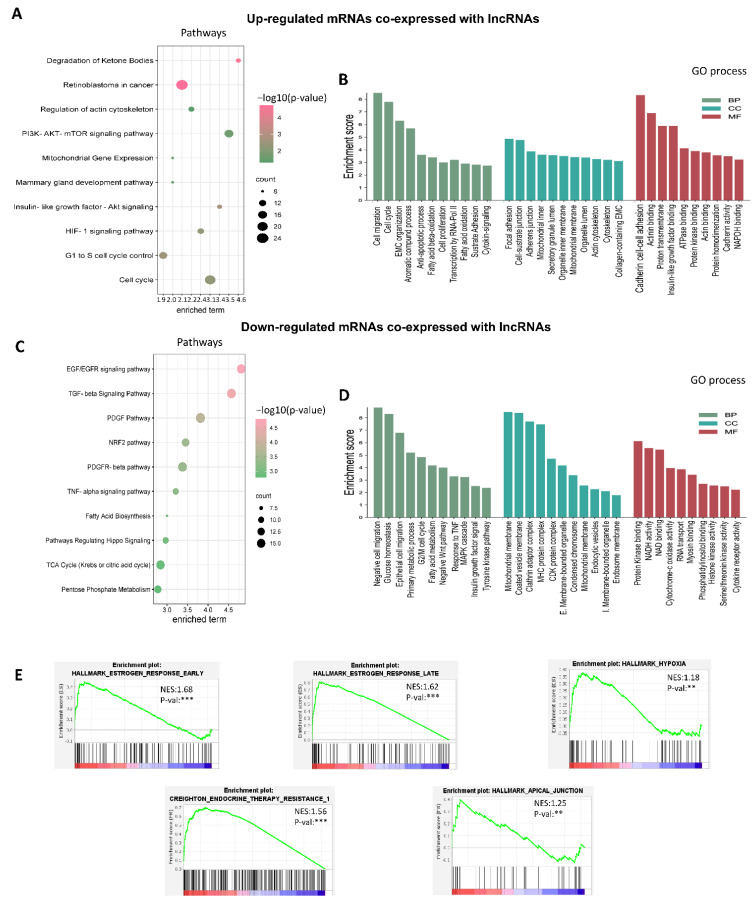
Analysis of biological functions related to mRNAs co-expressed with lncRNAs through Genetic Ontologies (GO), Pathways and Gene Set Enrichment Analysis. (**A**,**B**) Pathways and GO analysis of up-regulated mRNAs co-expressed with lncRNAs (**C**,**D**) Pathways and GO analysis of down-regulated mRNAs co-expressed with lncRNAs; BP (biological process), CC (cellular compartment), MF (molecular function) of GO analysis (**E**) GSEA analysis was done using mRNAs co-expressed with lncRNAs using datasets from both cancer hallmark and endocrine therapy resistance GSEA collections. Significant GSEA enrich processes (** *p* < 0.01, *** *p* < 0.001; NES ± 1.0).

Next, mRNAs co-expressed with lncRNAs were subjected to signaling pathways (Figure 4A–C) and GO analyses (Figure 4B–D). Signaling pathways such as EGFR and TGFB appeared enriched in the group of downregulated mRNAs and co-expressed with lncRNAs (Figure 4C). Interestingly, both signaling pathways have been linked to the activation of glycolysis and mitochondrial respiration [56,57]. These results could be related to enriched GO terms such as glucose homeostasis and mitochondrial membrane (Figure 4D).

GO analysis of up-regulated mRNAs indicated an enrichment mainly in the regulation of migration and the cell cycle, focal adhesion, the binding of cadherins involved in cell–cell adhesion and cell–substrate binding related to proteins that attach cells to specific compounds in the extracellular matrix (Figure 4B). These findings are consistent with previous reports indicating that 3D cell culture models influence the cell structure and cell–cell and ECM–cell interactions [58]. The activation of heterogeneous genetic expression programs, possibly linked to the multiple layers present in 3D structures, was observed in pathway analysis (Figure 4A, Supplementary Data S2). For instance, cell cycle inhibitors like CDKN1B, RFC5, SMAD4, and YWHAB, as well as cell cycle activators like CCNE2, MCM6, CDK2, and MCM2 were found as enriched in cell cycle processes. In addition, key essential signaling pathways for cancer development, including PI3K-AKT, HIF-1, and IGFR, were shown to be enriched in 3D cultures.

Gene set enrichment analysis (GSEA) of mRNA co-expression with lncRNAs was performed to investigate the biological pathways related to cancer hallmarks. GSEA generated an ordered list of genes related to cancer hallmarks by group based on co-expression network lncRNA/mRNA. Estrogen response hallmarks, both late (NES score 1.62) and early (NES score 1.68), were the most enriched hallmarks, as well as apical junctions (NES score 1.25) and hypoxia (NES score 1.38). Based on these findings, we hypothesize that our 3D culture model mimics an avascular primary tumor, where abnormal cell–cell interactions and a poor and hostile oxygen and nutritional profile are first generated [59].

On the other hand, a GSEA analysis was carried out to investigate the significance of therapy resistance in these 3D culture models. We employed a collection of genes associated to endocrine therapy resistance in breast cancers expressing estrogen receptor (ESR1) and ERBB2 (HER2) and identified a significant enrichment (NES 1.56). The mRNAs co-expressed with lncRNAs with the highest enrichment and that were overexpressed in 3D cultures were AFF3, OLFM1, DMKN, RIMS2, NAALADL2, STC2, HEY2, SULT2B1, and BCL2 (Figure 4E; Supplementary Data S3). Previous research has shown that over-expression of these genes contributes to resistance to chemotherapeutic treatments such as tamoxifen in breast cancer [60,61,62].

### 3.4. Co-Expression Networks between lncRNAs/mRNAs Could Regulate Key Processes for the Development and Maintenance of Cancer Hallmarks

**Figure 5:**
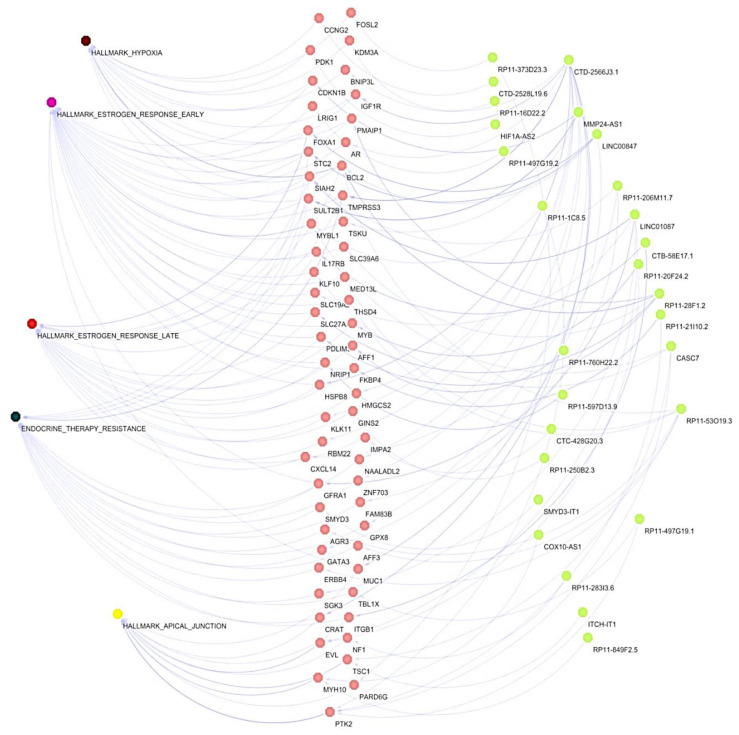
Co-expression lncRNAs/mRNAs networks common in BT-474 cell grown in 3D cultures and breast tumors (TCGA). The diagram shows how the cellular processes associated to cancer hallmarks, could be regulated by ncRNAs/mRNAs pairs that are common in BT-474 cultures and breast biopsies. Blue dot: lncRNAs; green dot: mRNAs; pink dot; multiple colors; cancer hallmarks and therapy resistance.

To better characterize the co-expression of lncRNAs/mRNAs pairs found modulated in BT-474 3D cultures, we compared them with the co-expression data of lncRNAs/mRNAs reported in TGCA from luminal B breast cancers patients (Figure 3A, Supplementary Data S1). A total of 764 common lncRNAs/mRNAs correlations were found between 3D cultures of BT-474 cell line and luminal B breast cancer samples from TCGA (Supplementary Data S4). In relation to hallmark cancer exacerbated in 3D cultures such as hypoxia, cell adhesion, response to estrogen, and therapy resistance (Figure 4E), we found 102 co-expressions of lncRNAs/mRNAs pairs, which belonged to 58 mRNAs and 27 lncRNAs present in both 3D cultures and TCGA (Figure 5). The co-expression network shows a marked enrichment in nodes of genes involved in response to estrogens, resistance to therapy, and interconnection between hallmarks through mRNAs participating in two or more processes, as observed for BCL2, SIAH2, and STC2, which connect estrogen responses, hypoxia, and resistance to therapy (Figure 5). 

Interestingly, the networks show how these interconnections are amplified through the participation of multiple lncRNAs. For example, CTD-2566J3.1 appears to be a master lncRNA gene by controlling most of the enriched processes from estrogen responses to apical junctions (Figure 5), through different mRNAs such as *IGF1R*, *BNIP3L*, *HSPB8*, *MUC1*, *TMPRSS3*, *RBM22*, *KLK11*, *HMGCS2*, *CRAT*, *GPX8* that participate in all enriched GSEA processes. *LINC01087* promotes the interconnection of early estrogens responses, hypoxia and therapy resistance through SIAH2, GFRA1, SGK3, EVL, and SLC27A. Also, LINC00847 and RP11-28F1.2 appear to be important regulators of key genes, such as *FOXA1*, *STC2*, *BCL2*, *GATA3, PMAIP1, SLC27A2,* and *SLC39A6,* respectively, promoting the activation of estrogen responses, hypoxia, apical junction, and therapy resistance.

**Figure 6:**
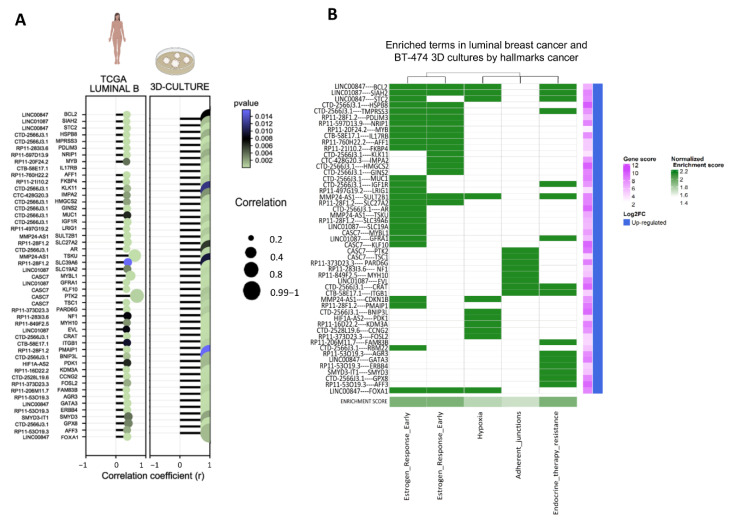
Co-expressed lncRNAs/mRNAs individual pairs in both 3D cultures and TCGA breast cancer (Lumina B). (**A**) Comparison of Pearson’s R values of lncRNAs/mRNAs pairs found in 3D cultures and luminal B breast cancer samples. (**B**) lncRNAs/mRNAs pairs in cancer hallmarks in co-expressed in 3D cultures and luminal B breast cancer.

Subsequently, as several mRNAs display interactions with diverse lncRNAs and GSEA-enriched terms, we sought to identify unique correlations of mRNAs with individual lncRNAs found in TCGA and 3D cultures using the highest positive R-values of Pearson’s correlations. Figure 6A shows the correlation coefficient found for each unique lncRNA/mRNA pair in both data sets (Figure 6A). In particular, we grouped unique pairs of mRNAs/lncRNAs to determine their participation in responses to estrogens, hypoxia, apical junctions, and resistance to therapy. Interestingly, several lncRNAs appear over-represented in each term (Figure 6B). As expected, CTD-2566J3.1 is one of the lncRNAs most frequently over-represented in early and late estrogen responses, as well as in resistance to therapy. *LINC00847*, on the other hand, is an over-represented lncRNA that could be also influence the early response to estrogens, hypoxia, and therapy resistance. Additionally, other over-represented lncRNAs such as *CASC7* and *LINC01087* are frequently co-expressed with mRNAs related to apical junctions, specifically through *PTK2*, *TSC1,* and *EVL*, as well as early and late estrogen response terms.

### 3.5. lncRNAs/mRNAs Pairs Related to Genetic Dependency of Cancer

Once the interactions between lncRNAs/mRNAs were characterized to be enriched in cancer hallmarks, their importance was determined through cancer genetic dependencies. A genetic dependency can be broadly defined as a gene that is required for the proliferation and/or survival of cancer cells. Inhibition of the gene or its protein product will block the growth of cancer cells or trigger cell death, as described in the Achilles project [37]. To determine whether there are mRNAs co-expressed with lncRNAs related to genetic dependency in cancer, Deepmap data from the Achilles project were used, which measure how individual genes can affect cell survival through RNAi or CRISPR-Cas experiments. The DEMETER score is a statistical summary of data from the Achilles Project that quantifies the essentiality scores of 17,098 genes. DEMETER suggested that a score below −0.5 represents depletion or considerable effect in cell proliferation and survival, while a score below −1 represents a strong effect on the proliferation and survival of tumor cells; that is, genes with a DEMETER score below −0.5 threshold can be considered essential for cell growth.

**Figure 7:**
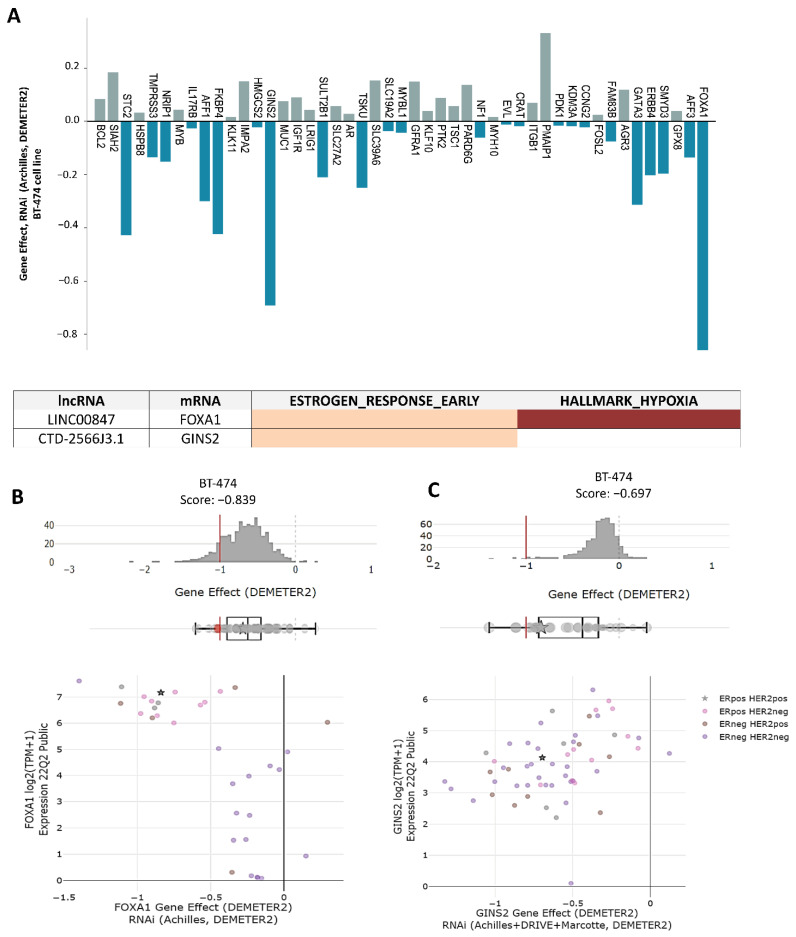
LINC00847-FOXA1 and CTD-2566J3.1 GINS2 are essential genes for proliferation and survival. (**A**) DEMETER score values for each mRNA reported in the co-expressed mRNAs/lncRNAs pairs of 3D cultures and TCGA data. (**B**,**C**) The DEMETER score is shown against the gene expression of *FOXA1* and *GINS2*; the stars represent BT-474 cell line and the color dots represent the breast cancer subtype of each cell line.

Our cancer genetic dependency analysis found two essential genes with a DEMETER score ≤ −0.5 in the BT-474 cell line, which correspond to *FOXA1* (DEMETER Score 0.83) and *GINS2* (DEMETER Score 0.69) (Figure 7A). Subsequently, DEMETER2 score comparisons were made in relation to gene expression in different molecular subtypes of breast cancer and we found that *FOXA1* is up-regulated and is an essential gene in luminal ER+ breast cancer cells. On the contrary, ER- molecular subtypes show a lower expression of *FOXA1* and a DEMETER score that does not reach the established threshold to consider it as an essential gene (Figure 7B). These data probably suggest that *FOXA1* is an essential and specific gene of the luminal-type of molecular subtypes in breast cancer, which is co-expressed with the lncRNA *LINC00847* and is related to the early response to estrogens and hypoxia. On the other hand, the data indicated that GINS2 is also an essential gene for the proliferation and survival of BT-474 cells, which was co-expressed with the lncRNA *CTD-2566J3.1*, to modulate the early response to estrogens. However, the DEMETER2 score did not show a molecular subtype-specific pattern (Figure 7C).

### 3.6. The lncRNAs, CTD-2566J3.1 and LINC00847, Are Associated with Overall Survival in Luminal B Breast Cancers

**Figure 8:**
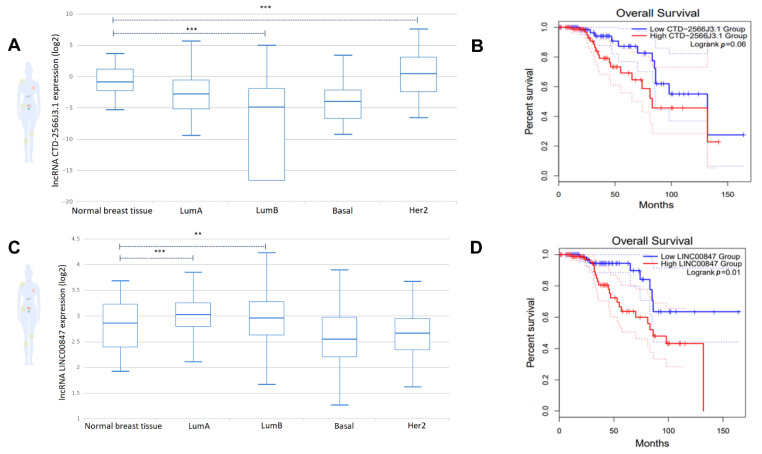
Expression of lncRNAs by molecular subtypes and impact on overall survival rates. (**A**) CTD-2566J3.1 gene expression in breast cancer molecular subtypes. (**B**) Kaplan-Meier survival curves for CTD-2566J3.1 associated with overall survival in luminal B breast cancer. The x-axis represents overall survival time (months) and the y-axis represents survival function. (**C**) LINC00847 gene expression in breast cancer molecular subtypes (**D**) Kaplan-Meier survival curves for LINC00847 associated with overall survival in luminal B breast cancer. The x-axis represents overall survival time (months) and the y-axis represents survival function. Student’s *t*-test (** *p* < 0.01, *** *p* < 0.001).

Due to the significant enrichment of terms potentially modulated by the lncRNAs CTD-2566J3.1, and LINC00847, their expression levels were examined in different molecular subtypes of breast cancer. Data showed that LINC00847 was upregulated in luminal molecular subtypes (Luminal A and Luminal B) compared to normal breast tissues. In addition, CTD-2566j3.1 expression was significantly upregulated in HER2-enriched tumors (Figure 8A–C), without changes in the other subtypes. Overall survival using Kaplan–Meier analyses in patients with luminal B-type breast cancer showed that up-regulation of both lncRNAs was correlated with poor prognosis, which was only statistically significant for LINC00847, since their up-regulation was negatively correlated with overall survival (log-rank *p* < 0.05) (Figure 8B–D).

### 3.7. Several mRNAs Co-Expressed with CTD-2566J3.1, and LINC00847 May Be Involved in Pathological Complete Response to Chemotherapy

Due to substantial enrichment of terms associated with therapy resistance and the negative relationship with overall survival of patients with luminal B breast cancer, we performed expression analyses and correlated with pathological complete response (PCR), defined as the absence of all signs of cancer in tissue samples obtained during surgery or biopsy after treatment with radiation or chemotherapy. Expression analyses and ROC curves were performed using mRNAs co-expressed with CTD-2566J3.1 and LINC00847 in samples from patients with luminal B-type breast cancer.

**Figure 9:**
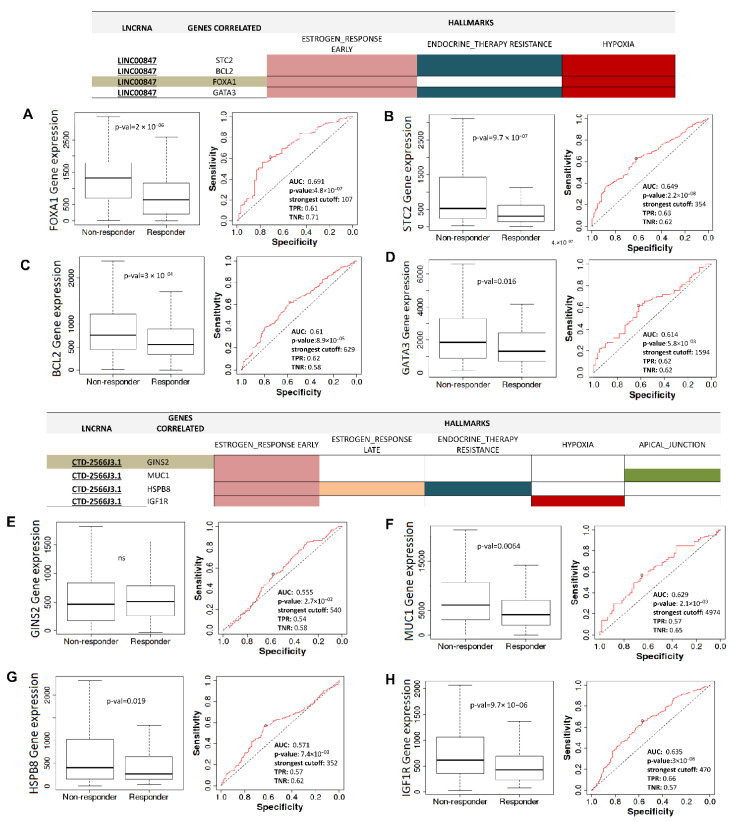
Pathological complete response related to lncRNAs/mRNAs pairs co-expressed in 3D cultures and breast cancer Luminal B tumors. (**A**–**D**) Gene expression of mRNAs related to LINC00847 in non-responder and responder and ROC values. (**E**–**H**) Gene expression of mRNAs related to CTD-2566J3.1 in non-responder and responder and ROC values.

The PCR analysis for the mRNAs co-expressed with the *LINC00847* showed that *FOXA1*, *STC2*, and *BCL2* were significantly over-expressed in patients without response to therapy (Figure 9A–C). Additionally, the ROC curve analysis revealed modest values of area under the curve (AUC > 0.6), being the highest value for FOXA1 (AUC 0.69) (Figure 9A), suggesting that, the low expression of FOXA1 can predict a PCR in 69% of patients after chemotherapy treatment. In addition, *MUC1* and *IGF1R* co-expressed with CTD-2566J3.1 had AUC > 0.6 and were found significantly over-expressed in patients without response to therapy, indicating that down-regulation of these mRNAs can modestly discriminate PCR in patients with luminal B breast cancer (Figure 9D–G).

## 4. Discussion

To better understand the molecular mechanisms that occur in an environment that mimics part of the tumor heterogeneity and microenvironment, we performed a microarray approach and found 473 dysregulated lncRNAs in 3D cultures from BT-474 cell line vs. 2D cultures. However, one of the most challenging aspects of researching lncRNA function is that they are not included in databases reporting their function, such as KEGG or GO, making it hard to know in which biological processes they are involved. To try to understand the function of lncRNAs, we adopted an indirect approach which is based on determining their co-expression with mRNAs by correlations. This indicates that co-expressed genes could be active simultaneously and participate in the same biological processes [63]. As expected, our GO analysis shows those up-regulated lncRNAs are co-expressed with mRNAs involved in cytoskeleton remodeling and cell adhesion, which have been observed in other reports of 3D cultures, where the organization of the extracellular matrix is one of the processes with the highest enrichment [33,64]. Interestingly, other studies carried out in 3D cultures of breast cancer have also found up-regulation of mitochondrial genes [33], agreeing in part with our results, where “mitochondrial gene expression” is up-regulated in 3D cultures of BT-474 (Figure 4A). Additionally, a similar biological process found in 3D cultures of BT-474 (Figure 4A), such as cell cycle, estrogen-mediated S-phase entry, cell cycle G1/S checkpoint regulation, and PI3K/AKT and PTEN signaling, have been reported up-regulated in 3D cultures of the MCF7 cell line [65]. On the other hand, a networks co-expression in spheroids of MCF7 cells found nodes for 63 lncRNA–mRNA co-regulated, and the main regulated biological network enriched in these nodes was the mTOR-signaling, MYC, and HIF-1 cascade [65]. In contrast, we found 102 co-expressions of lncRNAs/mRNAs pairs between 3D culture and luminal breast cancer related to estrogen responses, apical junctions, hypoxia, and resistance to therapy.

Additionally, lncRNAs could be active simultaneously with genes that regulate the tumor phenotype of three-dimensional cell models by enriching certain biological processes, such as cell cycle control. Cell cycle inhibitors including *CDKN1B*, *RFC5*, *SMAD4*, and *YWHAB*, as well as cell cycle activators like *CCNE2*, *MCM6*, *CDK2*, and *MCM2*, were shown to be enriched in cell cycle processes, G1/S, and retinoblastoma (Supplementary Data S2). We think that this mixture of co-expressed mRNAs is due in part to the diverse cell cycle distributions in different layers of a spheroid, ranging from quiescent to highly proliferative cells. Additionally, some important signaling pathways for the development and maintenance of tumorigenesis, such as PI3K-AKT, HIF-1, and IGFR, were enriched in 3D cultures. Furthermore, mRNAs/lncRNAs pairs with coordinated expression pattern are related to certain hallmarks of cancer, such as estrogen responses, hypoxia, and apical junctions, which are one of the major features found in 3D culture models of cancer with a modest degree of similarity with the correlation found in luminal B breast cancer. 

In relation to therapy resistance, endocrine therapy is the standard treatment for luminal-type breast cancers, which could be closely related to estrogens response, one of the hallmarks with the greatest enrichment score (Figure 4D). Estrogens play a key role in the proliferation and differentiation of healthy mammary epithelium, but they also contribute to the progression of breast cancer by promoting the growth of transformed cells [66] and, interestingly, the high expression of estrogen response genes has been related with the risk of recurrence of breast cancer [67]. Analysis of mRNAs co-expressed with lncRNAs suggested that endocrine therapy resistance could be a process involved in lncRNAs deregulation in luminal B breast cancer. Specifically, lncRNAs such as LINC01087, LINC00847, CTD-2566J3.1, and CTB-58E17.1 are found in co-expression with mRNAs up-regulated in 3D cultures related to endocrine therapy resistance, such as *AFF3*, *SULT2B1*, *GFRA1*, *SIAH2*, *STC2*, *MPRSS3*, *CRAT*, *BCL2,* and *ITGB1* (Figure 6B).

Following, we observed key lncRNAs/mRNAs pairs correlated are related to two or more cancer hallmarks, cancer genetic dependencies, overall survival, and pathological complete response. For example, LINC00847 has a similar expression pattern, with GATA-3/FOXA1/ERa axis related with estrogen responses, hypoxia, and therapy resistance in GSEA analysis. *LINC00847* has been shown to have an oncogenic effect, stimulating lung and liver cancer cell growth and metastasis [68,69]. STC2 is significantly up-regulated in hormone-positive breast cancers when compared with normal tissue, and a robust correlation with late recurrence in tamoxifen-treated patients has been found [70]. High levels of *BLC2* have also been linked to treatment resistance in ER+ breast tumors treated with tamoxifen [62]. Interestingly, *FOXA1* is described as a member of the forkhead family protein that facilitates chromatin binding and function of lineage-specific and oncogenic transcription factors like estrogen receptor (ER). *FOXA1* overexpression has been reported in luminal breast cancer and endocrine therapy resistance. These reports are consistent with the results obtained, where the co-expression of LINC00847/FOXA1 is reported both in data from patients with luminal B breast cancer and 3D organotypic cultures and is associated with the function of the response to estrogens, overall survival, and complete pathologic response. Interestingly, *GATA3* is another mRNA co-expressed with *LINC00847*. Some studies have highlighted an axis of positive regulation between GATA-3 and FOXA1 within the ERα pathway [71,72]. GATA-3 and FOXA1 interact with ERa, allowing transcription of almost 50% of genes induced by estrogen [72]. 

Multiple mRNAs, including *GINS2*, *MUC1*, *IGF1R*, *CRAT*, and *HSPB8,* were co-expressed with CTD-2566J3.1, which controls from the estrogen response to apical junctions. *MUC1* is abnormally up-regulated in estrogen receptor-positive breast cancer and produces tamoxifen resistance through receptor tyrosine kinases (RTK), B-catenin, and E-cadherin to mediate tumor growth control in response to tamoxifen [73]. Similarly, in luminal breast cancer, *IGF1R* is up-regulated and promoted antiestrogen resistance by activating PAK2/PIX [74]. Another study on tamoxifen resistance found that *HSPB8* expression is higher in MCF-7 cells resistant to tamoxifen compared to parent cells. Furthermore, increased *HSPB8* expression is linked to a worse outcome in ER+ breast cancer patients [75]. Finally, these findings show that 3D culture models are important pre-clinical tools with a high degree of similarity to tumoral tissue in relation to correlation of lncRNAs/mRNAs pairs. Using a co-expression approach, we identify different lncRNAs co-expressed with mRNAs related to responses to estrogens, hypoxia, apical junctions, and resistance to therapy. In conclusion, our data show that co-expressed lncRNAs/mRNAs pairs exhibit a high degree of similarity with those found in luminal B breast cancer patients, suggesting that they could be adequate pre-clinical tools to identify not only biomarkers related to endocrine therapy response and PCR, but to understand the biological behavior of cancer cells in 3D microenvironments, which point towards an important contribution of the roles of lncRNAs in organotypic 3D cultures.

## Figures and Tables

**Table 1:** Up-regulated lncRNAs in 3D cultures.

LncRNA	Fold Change	Function	Targets	Cancer Type	Reference
RP11-20F24.2	632.4	Metastasis	ANKRD30A	Normal breast tissues	[44]
THRA1/BTR	243.7	Unknown	Unknown		N/A
RP11-206M11.7	138.68	Unknown	Unknown		N/A
CTD-2566J3.1	115.75	Unknown	Unknown		N/A
UCA1	28.37	Hypoxia and apoptosis	HIF-1α, miR-206	Breast cancer/2D cell lines	[45]
LINC00672	27.32	Chemosensitivity	p53	Endometrial cancer/Xenograft mouse model	[46]
LINC01535	23.34	Proliferation, migration and invasion	miR-214/EZH2	Cervical cancer/Tumor samples and 2D cell lines	[47]
CTB-119C2.1	20.69	Unknown	Unknown		N/A
MIR924HG	19.66	Unknown	Unknown		N/A
PRRT3-AS1	19.41	Proliferation, apoptosis and autophagy	PPARγ	Prostate cancer/Tumor samples and 2D cell lines	[48]

**Table 2:** Down-regulated lncRNAs in 3D cultures.

LncRNA	Fold Change	Function	Targets	Cancer Type	Reference
XIST	−2786.08	Proliferation	AKT/HDAC3 PD-L1	Breast cancer/2D cell lines	[49]
RP11-782C8.3	−122.01	Unknown	Unknown		N/A
LINC00152	−78.28	Proliferation	miR-139-5p	Colorectal Cancer/Tumor samples and 2D cell lines	[50]
MIR4435-2HG	−35.98	Proliferation and migration	miR-206/YAP1	Colorectal Cancer/Tumor samples and 2D cell lines	[51]
CTD-2538C1.2	−32.1	Unknown	Unknown		N/A
LINC00857	−29.98	Proliferation and invasion.	CCNE1/CDK2	Lung Cancer/Tissues and 2D cell lines	[52]
RP11-425M5.7	−29.61	Unknown	Unknown		N/A
BLCAP-AS1	−25.26	Unknown	Unknown		N/A
RP11-383J24.6	−24.71	Unknown	Unknown		N/A
CH17-360D5.2	−24.61	Unknown	Unknown		N/A

## Data Availability

Microarray data is available at GSE206836 GEO database.

## Supplementary Materials

The following supporting information can be downloaded at: https://www.mdpi.com/article/10.3390/cells11213458/s1, Table S1: Pearson correlations in 3D cultures, Table S2: Terms pathway of Up/Down mRNAs correlated with lncRNAs, Table S3: GSEA enrichent term, Table S4: Correlations of mRNAs and lncRNAs in 3D culture and TCGA data from luminal B breast cancer patiens.
